# Synthesis of Marine α-Methoxylated Fatty Acid Analogs that Effectively Inhibit the Topoisomerase IB from *Leishmania donovani* with a Mechanism Different from that of Camptothecin

**DOI:** 10.3390/md11103661

**Published:** 2013-09-30

**Authors:** Néstor M. Carballeira, Nashbly Montano, Raquel Alvarez-Velilla, Christopher F. Prada, Rosa M. Reguera, Rafael Balaña-Fouce

**Affiliations:** 1 Department of Chemistry, University of Puerto Rico, PO Box 23346, San Juan 00931-3346, Puerto Rico; E-Mail: nashbly@hotmail.com; 2 Department of Biomedical Sciences, University of León, Campus de Vegazana s/n, León 24071, Spain; E-Mails: ralvv@unileon.es (R.A.-V.); rmregt@unileon.es (R.M.R.); rbalf@unileon.es (R.B.-F.); 3 Infectious Diseases Research Center of the CHUL of Québec and Laval University, Québec City, Québec G1V 4G2, Canada; E-Mail: Christopher.Fernandez@crchuq.ulaval.ca

**Keywords:** methoxylated fatty acids, *leishmania donovani*, 2-methoxy-5,9-eicosadienoic acid, topoisomerase IB

## Abstract

Sponges biosynthesize α-methoxylated fatty acids with unusual biophysical and biological properties and in some cases they display enhanced anticancer activities. However, the antiprotozoal properties of the α-methoxylated fatty acids have been less studied. In this work, we describe the total synthesis of (5*Z*,9*Z*)-(±)-2-methoxy-5,9-eicosadienoic acid (**1**) and its acetylenic analog (±)-2-methoxy-5,9-eicosadiynoic acid (**2**), and report that they inhibit (EC_50_ values between 31 and 22 µM) the *Leishmania donovani* DNA topoisomerase IB enzyme (*Ld*TopIB). The inhibition of *Ld*TopIB (EC_50_ = 53 µM) by the acid (±)-2-methoxy-6-icosynoic acid (**12**) was studied as well. The potency of *Ld*TopIB inhibition followed the trend **2** > **1** > **12**, indicating that the effectiveness of inhibition depends on the degree of unsaturation. All of the studied α-methoxylated fatty acids failed to inhibit the human topoisomerase IB enzyme (*h*TopIB) at 100 µM. However, the α-methoxylated fatty acids were capable of inhibiting an active but truncated *Ld*TopIB with which camptothecin (CPT) cannot interact suggesting that the methoxylated fatty acids inhibit *Ld*TopIB with a mechanism different from that of CPT. The diunsaturated fatty acids displayed low cytotoxicity towards *Leishmania infantum* promastigotes (EC_50_ values between 260 and 240 µM), but **12** displayed a better cytotoxicity towards *Leishmania donovani* promastigotes (EC_50_ = 100 µM) and a better therapeutic index.

## 1. Introduction

A selected group of marine sponges, such as *Calyx podatypa*, *Tropsentia roquensis* or *Higginsia tethyoides*, biosynthesize unusual α-methoxylated fatty acids with saturated, monounsaturated, and diunsaturated alkyl chains [1]. The most ubiquitous monounsaturated α-methoxylated fatty acids in the phospholipids of sponges are those with a Δ^6^ double bond, while among the diunsaturated α-methoxylated fatty acids the Δ^5,9^ double bonds predominate akin with the propensity of sponges to biosynthesize very long-chain Δ^5,9^ fatty acids [2,3]. More recently, the anticancer activity of this interesting group of fatty acids has received renewed attention, and there is substantial evidence indicating that α-methoxylation increases the anticancer properties of fatty acids by probably decreasing the fatty acid critical micelle concentration (CMC) [4].

The antiprotozoal activity of the marine α-methoxylated fatty acids has been less explored, but some interesting findings are now beginning to emerge, in particular promising inhibitory results against the DNA topoisomerase IB (*Ld*TopIB) from *Leishmania donovani*, the key parasite responsible for visceral leishmaniasis. *Ld*TopIB has a number of distinctive features that makes it a perfect therapeutic target. Unlike its human homologue of monomeric nature, the enzyme is a heterodimer and *Ld*TopIB is comprised of two subunits encoded by different genes, which are independently regulated [5]. When *Ld*TopIB is compared to the corresponding human enzyme (*h*TopIB), a low level of conservation of residues that comprise both proteins is observed. If sequence alignments between the two subunits of *Ld*TopIB and *h*TopIB are performed, it is found that the degree of similarity between the largest subunit of *Ld*TopIB and *h*TopIB is very low (45%) and even much lower (29%) when the small subunits are compared. These differences are important because it is just in these non-conserved regions where we find the residues responsible for drug sensitivity. A recent drug development effort targets these non-conserved regions by finding new drugs that can interfere with *Ld*TopIB without harming human cells. Camptothecin (CPT) and some of its derivatives such as gimatecan, topotecan, and irinotecan, to name just a few, have been investigated as *Ld*TopIB poisons [6].

While the saturated α-methoxylated fatty acids do not inhibit *Ld*TopIB, the monounsaturated α-methoxylated Δ^6^ fatty acids do display interesting inhibition of the enzyme. For example, our group recently synthesized the (±)-2-methoxy-6*Z*-heptadecenoic acid, a naturally occurring fatty acid from the sponge *Calyx podatypa*, and showed that it inhibits *Ld*TopIB with an EC_50_ of 41 ± 6 µM [7]. Moreover, we found that the synthetic alkynoic analog (±)-2-OMe-6-heptadecynoic acid displayed a better inhibition of the enzyme with an EC_50_ of 17 ± 1 µM [7]. These findings led us to conclude that an alkynoic α-methoxylated fatty acid could be a more effective inhibitor of *Ld*TopIB than an alkenoic α-methoxylated fatty acid provided that the carbon atoms in the acyl chain remain constant [7].

The Caribbean sponge *Erylus goffrilleri* is unusual in the sense that it biosynthesizes α-methoxylated Δ^5,9^ fatty acids [2]. Among these compounds, the (5*Z*,9*Z*)-(±)-2-methoxy-5,9-eicosadienoic acid (**1**) was identified in the phospholipids of *E. goffrilleri* together with other shorter-chain α-methoxylated Δ^5,9^ fatty acids [2]. Due to the low natural abundance of these α-methoxylated fatty acids in the sponge (ca. 0.3%), it was not possible to study their antiprotozoal or other related biological properties in the absence of a synthetic methodology that could provide enough quantities of fatty acids for biological screenings. In addition, the complete characterization of **1** was not possible. Therefore, in the present work we present the first total synthesis of **1** utilizing our previously developed methodology for this type of fatty acids [8], and show that **1** inhibits *Ld*TopIB with a mechanism different from that of CPT. In addition, taking advantage of the same synthetic route developed for **1**, and based on previous findings that alkynoic fatty acids are better inhibitors of *Ld*TopIB than alkenoic fatty acids [7], the unnatural analog (±)-2-methoxy-5,9-eicosadiynoic acid (**2**) was also synthesized expecting **2** to display a better inhibition of *Ld*TopIB than **1**.

## 2. Results and Discussion

### 2.1. Synthesis of the α-Methoxylated Fatty Acids **1** and **2**

**Scheme 1:**
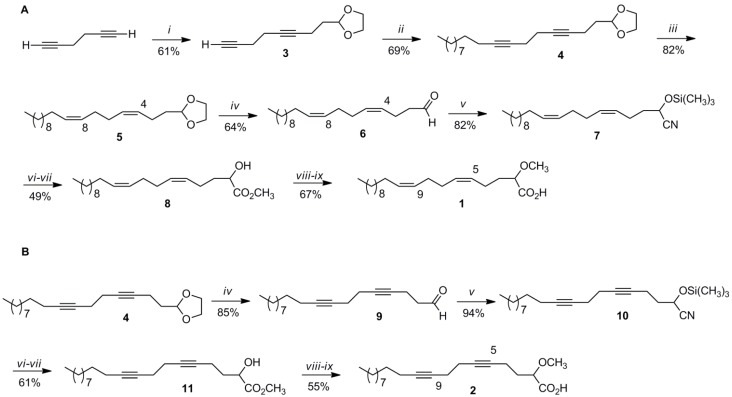
Synthesis of the α-methoxylated acids **1** (**A**) and **2** (**B**). (*i*) *n*-BuLi, THF-HMPA, 2-(2-bromoethyl)-1,3-dioxolane, −78 °C, 24 h; (*ii*) *n*-BuLi, THF-HMPA, 1-bromodecane, −10 °C, 24 h; (*iii*) H_2_, Lindlar/hexane, 24 h; (*iv*) HCl (conc.), acetone/H_2_O, 60 °C, 24 h; (*v*) TMSCN, Et_3_N, CH_2_Cl_2_, 0 °C, 3 h; (*vi*) HCl (conc.), 2-Me-THF, 60 °C, 24 h; (*vii*) HCl/MeOH, 35 °C, 3 h; (*viii*) NaH-CH_3_I, THF, 0 °C, 3 h; (*ix*) KOH/MeOH (1 M), heat, 2 h then HCl.

The synthesis of **1** followed the synthetic strategy previously developed in our group for this type of fatty acids and it is shown in Scheme 1 [8]. The synthesis started with the versatile starting material 1,5-hexadiyne (50% in pentane), which was coupled with 2-(2-bromoethyl)-1,3-dioxolane using *n*-BuLi in THF-HMPA at −78 °C, resulting in a 61% yield of the desired dioxolane **3** (Scheme 1). The next step called for coupling of dioxolane **3** with 1-bromodecane using similar reaction conditions as the first step resulting in the dioxolane **4** in a 69% yield. In order to install the correct stererochemistry for the two *cis* double bonds required for the final product, dioxolane **4** was hydrogenated under Lindlar conditions in hexane affording **5** (82% yield). The 100% *cis* stereochemistry for the two double bonds in **5** was confirmed by ^13^C NMR spectroscopy and capillary gas chromatography-mass spectrometry (GC-MS). In ^13^C NMR spectroscopy, the allylic carbon resonances are strongly dependent upon the stereochemistry of the adjacent double bonds and there is a significant difference (around 5 ppm) between the allylic carbon resonances (around 32 ppm) adjacent to a *trans* double bond and the allylic carbon resonances (around 27 ppm) adjacent to a *cis* double bond [9]. With the GC-MS analysis we were able to confirm that we only obtained the *cis*, *cis* product and no other combination of double bond stereochemical alternatives such as, for example, *cis*, *trans*. Removal of the dioxolane in **5** was most conveniently achieved under acidic conditions (HCl) using acetone/water (1:1) as solvent and heating at 60 °C for 24 h, resulting in a 64% yield of dienal **6**. Aldehyde **6** was then reacted with trimethylsilyl cyanide (TMSCN) in dichloromethane and catalytic amounts of triethylamine at 0 °C, yielding the nitrile **7** in an 82% yield. Nitrile **7** was then transformed into the desired methyl ester **8** in two subsequent steps. First, acid hydrolysis of **7** in 2-methyltetrahydrofuran (2-MeTHF) at 60 °C for 24 h afforded the (5*Z*,9*Z*)-(±)-2-hydroxy-5,9-eicosadienoic acid, which was esterified in HCl/methanol, without isolation, resulting in the desired methyl ester **8** in a combined 49% yield for the last two steps. The final acid **1** was obtained in two more steps. First, methylation of **8** with sodium hydride and methyl iodide in THF, afforded the methyl (5*Z*,9Z)-(±)-2-methoxy-5,9-eicosadienoate and after saponification in 1M KOH/ethanol and final purification the expected acid **1** was obtained in a combined 67% yield for the last two steps. The overall yield for this nine-step synthetic sequence was 5.9%. A GC-MS co-injection of methyl (5*Z*,9Z)-(±)-2-methoxy-5,9-eicosadienoate with the fatty acid methyl ester (FAME) mixture from *E. goffrilleri* confirmed that we synthesized the same α-methoxylated dienoic fatty acid as the naturally occurring fatty acid, thus corroborating the structure of the natural fatty acid as well as the stereochemistry of the two *cis* double bonds [2]. We are also reporting, for the first time, the complete spectral data for **1**.

Taking advantage of the previous synthetic sequence, acid **2** was also prepared by utilizing dioxolane **4** as the key intermediate. Removal of the dioxolane in **4** was also possible under acidic conditions (HCl) using acetone/water (1:1) as solvent and heating at 60 °C for 24 h, resulting in an 85% yield of diynal **9**. Similar to the synthetic procedure described above, diynal **9** was reacted with TMSCN in dichloromethane and catalytic amounts of triethylamine at 0 °C, resulting in a 94% yield of nitrile **10**. Diynonitrile **10** was then transformed into the desired methyl ester **11** in two steps. First, acid hydrolysis of **10** in 2-MeTHF at 60 °C for 24 h afforded the (±)-2-hydroxy-5,9-eicosadiynoic acid, which was esterified in HCl/methanol, without isolation, resulting in the desired methyl ester **11** in a combined 61% yield for the last two steps. Methylation of **11** with sodium hydride and methyl iodide in THF afforded the methyl (±)-2-methoxy-5,9-eicosadiynoate, and after saponification in 1M KOH/ethanol and final purification, the desired acid **2** was obtained in a combined 55% yield for the last two steps. The overall yield for this eight-step synthetic sequence was 11.2%.

### 2.2. Inhibition of LdTopIB by Acids **1** and **2**

**Figure 1:**
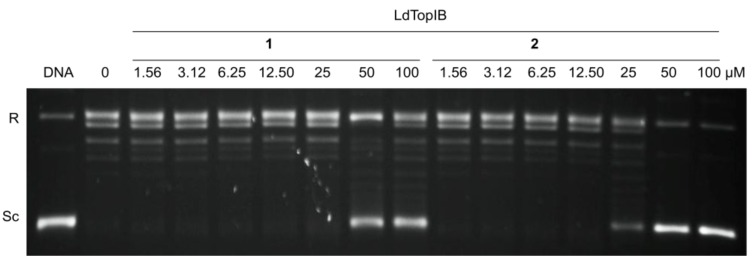
Comparison of the inhibition of the relaxation activity of recombinant *Ld*TopIB by **1** (left) and **2** (right). One unit of recombinant *Ld*TopIB was assayed in a plasmid DNA relaxation assay for 30 min at 37 °C in the presence of 1.56–100 µM of either acids **1** or **2**. Reaction products were resolved in agarose gel and subsequently visualized by ethidium bromide staining. The relative position of the negatively supercoiled DNA substrate is indicated by Sc, R is the relaxed DNA, whereas the ladder of relaxed DNA topoisomer bands is shown in between. Reactions were stopped with a mixture of 1% SDS and 6.1 µg of proteinase K. Lane 1 contains 0.5 µg of pSK plasmid DNA and lane 2, indicated by a 0, is 10% DMSO.

**Figure 2:**
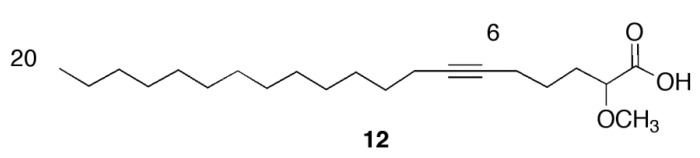
Structure of the (±)-2-methoxy-6-icosynoic acid (**12**).

Based on our previous results with the α-methoxylated Δ^6^ fatty acids [7], we concentrated on the inhibition of *Ld*TopIB by acids **1** and **2** so as to be able to compare the effect of a Δ^6^ unsaturation *versus* a Δ^5,9^ diunsaturation on the degree of inhibition of *Ld*TopIB (Figure 1, Table 1). As a mode of comparison, the inhibition of *Ld*TopIB by the (±)-2-methoxy-6-icosynoic acid (**12**) (Figure 2) was also investigated since it has the same carbon chain length as acids **1** and **2** and its synthesis was recently described [4]. As explained in the Introduction, *Ld*TopIB is a worthwhile target to study since recent strategies against leishmania take advantage of the structural differences between *Ld*TopIB and *h*TopIB, and because the unorthodox heterodimeric TopIB of kinetoplastid parasites, such as *Ld*TopIB, can be used for the development of novel drugs aimed at *Ld*TopIB without interfering with the host [10]. For this reason, the inhibition of both *Ld*TopIB and *h*TopIB by acids **1** and **2** was examined. As predicted, **2** was the most efficient inhibitor of *Ld*TopIB with an EC_50_ = 22 ± 1 μM followed by **1** with an EC_50_ = 31 ± 2 μM and finally **12** with an EC_50_ = 53 ± 3 μM (Table 1). Therefore, the effectiveness of *Ld*TopIB inhibition followed the order **2** > **1** > **12** and the data seems to support our hypothesis that as the degree of unsaturation increases, the inhibition of *Ld*TopIB by the fatty acid increases as well.

**Table 1:** Inhibition of the relaxation activities of *Ld*TopIB and *h*TopIB by the studied fatty acids and camptothecin (CPT) (µM).

Compounds	*Ld*TopIB EC_50_	*h*TopIB EC_50_
**1**	31 ± 2	>100
**2**	22 ± 1	>100
**12**	53 ± 3	>100
CPT	0.7 ± 0.1	2 ± 1

**Figure 3:**
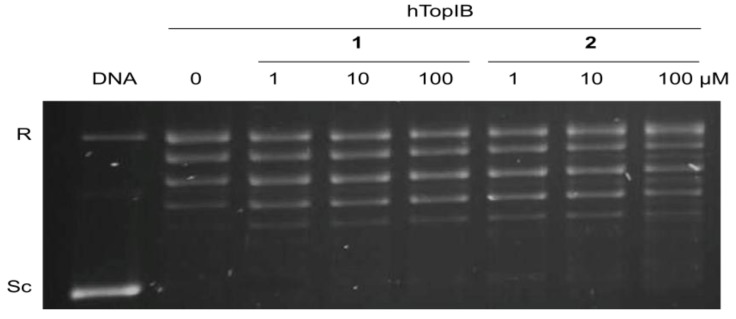
Comparison of the inhibition of the relaxation activity of *h*TopIB by **1** (left) and **2** (right). One unit *h*TopIB was assayed in a plasmid DNA relaxation assay for 30 min at 37 °C in the presence of 1–100 µM of either acids **1** or **2**. Reaction products were resolved in agarose gel and subsequently visualized by ethidium bromide staining. The relative position of the negatively supercoiled DNA substrate is indicated by Sc, R is the relaxed DNA, whereas the ladder of relaxed DNA topoisomer bands is shown in between. Reactions were stopped with a mixture of 1% SDS and 6.1 µg of proteinase K. Lane 1 contains 0.5 µg of pSK plasmid DNA and lane 2, indicated by a 0, is 10% DMSO.

In the latter experiment, the inhibition of *h*TopIB by acids **1** and **2** was also compared to the inhibition observed for *Ld*TopIB and the results are shown in Table 1 and Figure 3. While all of the α-methoxylated fatty acids studied herein were able to inhibit *Ld*TopIB at concentrations between 53 and 22 μM, they were not effective against *h*TopIB (EC_50_ > 100 μM). These results, once again, demonstrate that it can be possible to preferentially inhibit *Ld*TopIB without inhibiting *h*TopIB, a finding that could have medicinal applications. It is evident that *Ld*TopIB is more sensitive to inhibition by the α-methoxylated fatty acids than *h*TopIB.

**Figure 4:**
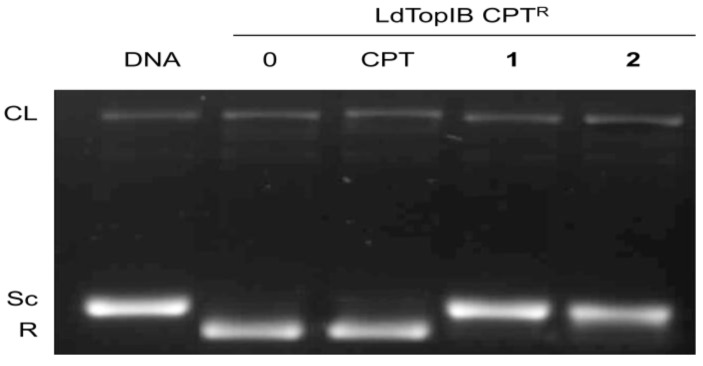
Methoxylated fatty acids inhibit *Ld*TopIB by a CPT-independent mechanism. CPT-resistant *Ld*TopIB CPT^R^ [12,13] was assayed in the presence of DMSO (lane 0), 100 μM CPT, 100 μM acid **1**, and 100 μM acid **2**. Samples were incubated for 4 min at 25 °C, stopped with 1% SDS and digested for one extra hour at 37 °C in the presence of 1 mg/mL proteinase K. DNA was extracted with one volume of phenol-chloroform and samples were run on a 1% agarose gel containing ethidium bromide to a final concentration of 40 μg/mL in order to separate supercoiled and relaxed DNA. The nicked band corresponds to the stabilized cleavage complexes. The results are representative of three independent trials.

To further study the mechanism by which the α-methoxylated fatty acids inhibit *Ld*TopIB we explored if these acids inhibit the enzyme with a mechanism similar or different to that of CPT, a well-known topoisomerase I inhibitor [11]. Towards this purpose we carried an assay whereby instead of using the wild type *Leishmania* enzyme we used a doubly truncated enzyme [*Ld*TopIB CPT^R^ (*Ld*TopIL^1−561/S175end^)] from which the sections responsible for interacting with CPT were removed and yet the enzyme still retained TopIB activity [12,13]. In Figure 4, we can see that CPT (at levels of 100 µM) is not able to inhibit the activity of *Ld*TopIB CPT^R^ nor has it the ability to increase the intensity of the CL band. However, we can also observe in Figure 4 (lanes 4 and 5) that both acids **1** and **2** are able to completely inhibit *Ld*TopIB CPT^R^ as judged by the band corresponding to the supercoiled (Sc) DNA. Therefore, this experiment clearly demonstrates that acids **1** and **2** inhibit the *Ld*TopIB-mediated DNA relaxation by a complete different mechanism as CPT.

Despite the fact that the exact mechanism by which fatty acids inhibit *Ld*TopIB still remains elusive, the best explanation to date, as we have hypothesized in previous studies, is for the α-methoxylated fatty acids to be interacting with *Ld*TopIB by binding in a region close to the topoisomerase active site and either inhibiting the enzyme binding to DNA or blocking the cleavage reaction step [14].

### 2.3. Toxicity of Acids **1**, **2**, and **12** towards *L. infantum* and *L. donovani*

As a logical next step after the *Ld*TopIB inhibitory studies, the toxicity of acids **1** and **2** towards *L. infantum*, which is closely related to *L. donovani*, was studied to determine if the inhibitory effects towards *Ld*TopIB translates into toxicity towards *L.* *infantum* promastigotes. Both acids **1** and **2** were weakly toxic to *L. infantum* promastigotes with EC_50_ values between 260 and 240 µM (Table 2). However, acid **2** was slightly more toxic to *L. infantum* than **1**, a finding that seems to correlate with the *Ld*TopIB studies. In a separate experiment, it was also found that acid **12** was more effective towards *L. donovani* promastigotes with an EC_50_ of 100 µM. From the *in vitro* studies we can conclude that the α-methoxylated fatty acids **1** and **2** are weakly toxic to *Leishmania* promastigotes, but surprisingly, **12** displayed the best therapeutic index, as it was more toxic to *L. donovani* promastigotes and less toxic to murine macrophages (Table 2). Murine macrophages were chosen as reference for this study since *Leishmania* spp. normally infects macrophages and in macrophages is where *Leishmania* promastigotes are transformed into *Leishmania* amastigotes. From these studies we can conclude that **12** displays the best correlation between the enzyme inhibitory studies and the parasite growth inhibition data with only a two-fold difference between the enzyme inhibitory EC_50_ (Table 1) and the toxicity IC_50_ (Table 2).

**Table 2:** Toxicities of the studied fatty acids and CPT towards *L. infantum* and murine macrophages (µM).

Compounds	*L. infantum* EC_50_	Murine Macrophages BALB/c IC_50_	Therapeutic Index (IC_50_/EC_50_)
**1**	260 ± 20	110 ± 10	0.4
**2**	240 ± 10	90 ± 20	0.4
**12**	100 ± 10 ^a^	>100	>1
CPT	1.1 ± 0.1	0.6 ± 0.1	0.5

^a^ IC_50_ (µM) against *L. donovani* promastigotes.

## 3. Experimental Section

### 3.1. Instrumentation

^1^H NMR (300 or 500 MHz) and ^13^C NMR (75 or 125 MHz) were either recorded on a Bruker DPX-300 or a Bruker DRX-500 spectrometer. ^1^H NMR chemical shifts are reported with respect to internal (CH_3_)_4_Si, ^13^C NMR shifts are reported in part per million relative to CDCl_3_ (77.0 ppm). GC/MS analysis were recorded at 70 eV using either a Hewlett Packard 5972A MS Chem Station or an Agilent 5975C MS Chem Station coupled to an Agilent 7890A. Both GC were equipped with a 30 m × 0.25 mm special performance capillary column (HP-5MS) of polymethyl siloxane crosslinked with 5% phenyl methylpolysiloxane. IR spectra were recorded on a Spectrum One FT-IR Spectrometer (PerkinElmer). High-resolution mass spectra data was performed at the Emory University Mass Spectrometry Center on a Thermo LTQ-FTMS using APCI as the probe.

### 3.2. Synthesis of (5*Z*,9*Z*)-(±)-2-Methoxy-5,9-eicosadienoic Acid (**1**)

#### 3.2.1. 2-(3,7-Octadiynyl)-1,3-dioxolane (**3**)

It was as obtained as a colorless oil in a 61% yield from the reaction of 3.7 mL (19.23 mmol) of 1,5-hexadiyne in 40 ml of dry THF with 5.1 mL (55.47 mmol) of *n*-BuLi (2.5 M in hexane) at −78 °C and 1.5 mL (12.85 mmol) of 2-(2-bromoethyl)-1,3-dioxolane following the procedure already described and with identical spectral data as previously reported [7].

#### 3.2.2. 2-(Octadeca-3,7-diynyl)-1,3-dioxolane (**4**)

To a stirred solution of **3 (**1.39 g, 7.81 mmol) in dry THF (13 mL) and under argon was added 2.1 mL (22.8 mmol) of *n*-BuLi (2.5 M) in hexanes at −10 °C followed by 45 min of stirring. After this time the temperature was lowered to −70 °C and 3.1 mL of HMPA was added to the reaction mixture followed by 2.7 mL (13.01 mmol) of 1-bromodecane, and stirring for 24 h. Then, the reaction mixture was quenched with water. The organic product was extracted with brine solution (2 × 15 mL), diethyl ether (2 × 15 mL), dried over MgSO_4_, filtered, and evaporated *in vacuo*. The product was purified using silica gel column chromatography eluting with hexane/ether (9:1). Dioxolane **4** (1.71 g, 5.37 mmol) was obtained as colorless oil for a 69% yield.

^1^H (CDCl_3_, 300 MHz) δ (ppm) 4.95 (1H, t, H-2), 3.95–3.81 (4H, m, -OCH_2_-), 2.30 (4H, m,), 2.25 (2H, m), 2.11 (2H, t, H-3), 1.81 (2H, m), 1.43 (2H, m), 1.26 (14H, brs, -CH_2_-), 0.86 (3H, t, -CH_3_); ^13^C (CDCl_3_, 75 MHz) δ (ppm) 103.3 (d, C-2), 81.2 (s), 79.8 (s), 79.1 (s), 78.6 (s), 64.9 (t), 33.2 (t), 31.9 (t), 29.7 (t), 29.5 (t), 29.3 (t), 29.1 (t), 29.0 (t), 28.8 (t), 22.7 (t), 19.4 (t), 19.3 (t), 18.8 (t), 14.1 (q, -CH_3_), 13.7 (t); GC/MS (70 eV) *m/z* (relative intensity): 318 (M^+^, 1), 303 (1), 290 (2), 289 (5), 273 (1), 235 (1), 191 (6), 177 (2), 163 (5), 139 (55), 105 (10), 99 (23), 86 (12), 73 (100); HRMS (APCI) Calcd for C_21_H_35_O_2_ [M + H]^+^ 319.2632, found 319.2630.

#### 3.2.3. 2-[(3*Z*,7*Z*)-Octadeca-3,7-dien-1-yl]-1,3-dioxolane (**5**)

Into a 50-mL two-necked round-bottomed flask containing Lindlar’s catalyst (0.64 g) was added a solution of **4** (0.27 g, 0.86 mmol) in dry hexane (8.6 mL) and catalytic amounts of quinoline. The reaction mixture was stirred under H_2_ for 24 h, filtered, and the solvent removed *in vacuo*. The product was purified under vacuum distillation (Kugelrohr) by removing impurities and quinoline at 130 °C/3 mmHg. The dioxolane **5** (0.23 g, 0.70 mmol) was obtained as colorless oil in an 82% yield.

IR (NaCl) ν_max_: 3005, 2924, 2854, 1456, 1408, 1212, 1140, 1040, 944, 723 cm^−1^; ^1^H (CDCl_3_, 300 MHz) δ (ppm) 5.45–5.39 (m, 4H, -CH=CH-), 4.85 (1H, t, H-2), 3.97–3.85 (4H, m, -OCH_2_-) 2.17 (2H, m), 2.09 (2H, m), 2.00 (2H, m), 1.71 (2H, m), 1.29 (16H, brs, -CH_2_-), 0.87 (3H, t, -CH_3_); ^13^C (CDCl_3_, 75 MHz) δ (ppm) 130.5 (d), 130.0 (d), 129.0 (d), 128.9 (d), 104.1 (d, C-2), 64.9 (t), 33.8 (t), 31.9 (t), 29.7 (t), 29.6 (t), 29.5 (5), 29.3 (t), 29.2 (t), 27.3 (t), 22.7 (t), 21.4 (t), 14.1 (q, -CH_3_); GC/MS (70 eV) *m/z* (relative intensity): 322 (M^+^, 1), 321 (1), 279 (3), 265 (1), 239 (1), 195 (4), 155 (6), 141 (32), 128 (22), 119 (8), 99 (73), 86 (35), 81 (18), 79 (29), 73 (100), 69 (15), 67 (23), 55 (22); HRMS (APCI) Calcd for C_21_H_39_O_2_ [M + H]^+^ 323.2945, found 323.2943.

#### 3.2.4. (4*Z*,8*Z*)-Nonadeca-4,8-dienal (**6**)

To a stirred solution of **5** (0.20 g, 0.62 mmol) in acetone/water (1.6 mL each) was added 0.6 mL of HCl (conc.) followed by reflux at 60 °C for 24 h. Then, the reaction mixture was extracted with diethyl ether (1 × 15 mL), water (1 × 15 mL), dried over MgSO_4_, filtered, and evaporated *in vacuo* affording aldehyde **6** (0.11 g, 0.40 mmol) in 64% yield as colorless oil.

IR (NaCl) ν_max_: 3418, 3007, 2925, 2855, 1715 (C=O), 1464, 1377, 1258, 1051, 722 cm^−1^; ^1^H (CDCl_3_, 300 MHz) δ (ppm) 9.77 (1H, t, CHO), 5.43-5.35 (4H, m, -CH=CH-), 2.48 (2H, m), 2.37 (2H, m), 2.09 (4H, m), 2.02 (2H, m), 1.26 (16H, brs, -CH_2_-), 0.87 (3H, t, -CH_3_); ^13^C (CDCl_3_, 75 MHz) δ (ppm) 202.2 (d, C-2), 131.0 (d), 130.7 (d), 128.7 (d), 127.5 (d), 43.8 (t), 31.9 (t), 29.7 (t), 29.63 (t), 29.60 (t), 29.3 (t), 27.3 (t), 27.1 (t), 22.7 (t), 20.1 (t), 14.1 (q, -CH_3_); GC/MS (70 eV) *m/z* (relative intensity): 278 (M^+^, 1), 260 (1), 249 (1), 137 (11), 134 (11), 123 (10), 119 (13), 111 (13), 109 (14), 97 (90), 95 (29), 83 (69), 81 (36), 79 (74), 69 (100), 67 (69), 57 (40), 55 (94); HRMS (APCI) Calcd for C_19_H_35_O [M + H]^+^ 279.2682, found 279.2682.

#### 3.2.5. (5Z,9Z)-(±)-2-Trimethylsilyloxy-5,9-eicosadienonitrile (**7**)

To a stirred solution of aldehyde **6** (0.11 g, 0.38 mmol) in dry CH_2_Cl_2_ (5.0 mL) at 0 °C was added trimethylsilyl cyanide (TMSCN) (0.8 mL, 0.57 mmol) and catalytic amounts of triethylamine. The mixture was stirred under argon for 3 h. Then, the solvent was removed *in vacuo*, the crude mixture washed with water (2 × 10 mL), extracted with diethyl ether (2 × 10 mL), dried over MgSO_4_, filtered, and after solvent removal **7 (**0.12 g, 0.31 mmol) was obtained as a yellow oil in 82% yield.

IR (NaCl) ν_max_: 3800, 2925, 2854, 2251 (CN), 1655, 1437, 1254, 1078, 846, 722 cm^−1^; ^1^H (CDCl_3_, 300 MHz) δ (ppm) 5.45–5.31 (4H, m, -CH=CH-), 4.39 (1H, t, H-2), 2.22 (2H, m), 2.11 (4H, m), 2.00 (2H, m), 1.83 (2H, m), 1.25 (12H, brs, -CH_2_-), 0.87 (3H, t, -CH_3_), 0.19 (9H, s, -OSi(CH_3_)_3_); ^13^C (CDCl_3_, 75 MHz) δ (ppm) 131.4 (d), 130.7(d), 128.7 (d), 127.5 (d), 120.0 (s, C-1), 60.8 (d, C-2), 36.1 (t), 31.9 (t), 29.7 (t), 29.6 (t), 29.5 (t), 29.3 (t), 29.2 (t), 27.4 (t), 27.3 (t), 27.1 (t), 22.7 (t), 22.3 (t), 14.1 (q, -CH_3_), -0.39 (q, -OSi(CH_3_)_3_); GC/MS (70 eV) *m/z* (relative intensity): 377 (M^+^, 17), 362 (16), 350 (11), 349 (30), 286 (4), 234 (10), 208 (14), 160 (12), 155 (18), 142 (29), 128 (40), 119 (25), 116 (73), 106 (33), 97 (43), 83 (53), 81 (41), 80 (77), 79 (67), 75 (36), 73 (100), 69 (59), 67 (62), 57 (36), 55 (74); HRMS (APCI) Calcd for C_23_H_44_O_2_NSi [M + H]^+^ 378.3187, found 378.3187.

#### 3.2.6. Methyl (5*Z*,9*Z*)-(±)-2-Hydroxy-5,9-eicosadienoate (**8**)

To a stirred solution of **7** (0.12 g, 0.34 mmol) in 2-MeTHF (5.1 mL) was added concentrated HCl (2.0 mL). The reaction mixture was stirred at 60 °C for 24 h. After this time, the crude was washed with water (2 × 10 mL), extracted with diethyl ether (2 × 10 mL), dried over MgSO_4_, filtered, and evaporated *in vacuo*. After purification by Kugelrohr distillation at 140 °C/3 mmHg, the (5*Z*,9*Z*)-(±)-2-hydroxy-5,9-eicosadienoic acid was obtained which was esterified in 20.0 mL of methanol by adding HCl (conc.), and stirring for 3 h at 35 °C. After methanol removal *in vacuo*, the crude was washed with water (2 × 10 mL), ether (2 × 10 mL), dried over MgSO_4_, filtered, and concentrated by evaporation *in vacuo*. The methyl ester was purified using silica gel column chromatography eluting with hexane/ether (7:3). Methyl ester **8** (0.06 g, 0.17 mmol) was obtained as colorless oil in a 49% overall yield for the last two steps.

IR (NaCl) ν_max_: 3501, 2926, 2855, 1740 (C=O), 1456, 1436, 1362, 1199, 1127, 807, 722 cm^−1^; ^1^H (CDCl_3_, 300 MHz) δ (ppm) 5.45–5.31 (4H, m, -CH=CH-), 4.19 (1H, m, H-2), 3.79 (3H, s, -OCH_3_), 2.19 (2H, m), 2.09 (4H, m), 2.01 (2H, m), 1.88 (2H, m), 1.27 (12H, br s, -CH_2_-), 0.87 (3H, t, -CH_3_); ^13^C (CDCl_3_, 75 MHz) δ (ppm) 175.8 (s, C-1), 130.7 (d), 130.5 (d), 128.9 (d), 128.3 (d), 69.9 (d, C-2), 52.5 (q, -OCH_3_), 31.9 (t), 29.6 (t), 29.5 (t), 29.3 (t), 29.0 (t), 27.3 (t), 25.3 (t), 22.7 (t), 14.1 (q, -CH_3_); GC/MS (70 eV) *m/z* (relative intensity): 338 (M^+^, 1), 322 (1), 307 (1), 295 (1), 279 (3), 261 (6), 213 (6), 165 (11), 157 (13), 139 (13), 123 (11), 109 (20), 97 (100), 90 (74), 83 (32), 81 (40), 79 (65), 69 (48), 67 (51), 57 (21), 55 (54); HRMS (APCI) Calcd for C_21_H_39_O_3_ [M + H]^+^ 339.2894, found 339.2897.

#### 3.2.7. (5*Z*,9*Z*)-(±)-2-Methoxy-5,9-eicosadienoic acid (**1**)

To a stirred solution of NaH (0.01 g, 0.50 mmol) in dry THF (3.0 mL) under argon was added a solution of **8** (0.05 g, 0.14 mmol) in dry THF (3.0 mL). The reaction mixture was stirred at rt for 10 min, and then methyl iodide (0.03 mL, 0.48 mmol) was added dropwise at 0 °C, followed by 3 h stirring. After that, HCl (conc.) was added to the solution until the pH was acidic. The crude was extracted with diethyl ether (2 × 10 mL), dried over MgSO_4_ and evaporated *in vacuo*. The product was purified using silica gel column chromatography first eluting with hexane/ether (9:1) and then with ether affording the methyl (5*Z*,9*Z*)-(±)-2-methoxy-5,9-eicosadienoate. To obtain **1**, a solution of KOH/ethanol (1 M) (20.0 mL) and the methoxylated methyl ester was stirred for 2 h at 60 °C. After this time the solvent was removed *in vacuo* and hexane (5.0 mL) and 5.0 mL of 6M HCl was added to the solution. The crude product was washed with water (1 × 10 mL), diethyl ether (2 × 10 mL), dried over MgSO_4_, filtered, and the ether evaporated *in vacuo*. The final product was purified using Florisil^®^ (activated magnesium silicate) column chromatography eluting with diethyl ether affording **1** (0.032 g, 0.09 mmol) as an oil for a 67% yield.

IR (NaCl) ν_max_: 3500–2500 (-OH), 3006, 2925, 2854, 1706 (C=O), 1463, 1378, 1200, 1125, 722 cm^−1^; ^1^H (CDCl_3_, 500 MHz) δ (ppm) 5.45–5.31 (4H, m, -CH=CH-), 3.81 (1H, t, H-2), 3.44 (3H, s, -OCH_3_), 2.21 (2H, m), 2.10 (4H, m), 2.01 (2H, m), 1.27 (16H, brs, -CH_2_-), 0.87 (3H, t, -CH_3_); ^13^C (CDCl_3_, 125 MHz) δ (ppm) 177.3 (s, C-1), 130.9 (d, C-10), 130.6 (d, C-6), 128.9 (d, C-9), 128.1 (d, C-5), 79.5 (d, C-2), 58.3 (q, -OCH_3_), 31.9 (t), 29.7 (t), 29.69 (t), 29.64 (t), 29.5 (t), 29.3 (t), 27.3 (t), 27.29 (t), 27.24 (t), 22.7 (t), 22.6 (t), 14.1 (q, -CH_3_); HRMS (APCI) Calcd for C_21_H_37_O_3_ [M + H]^+^ 337.2748, found 337.2743.

### 3.3. Synthesis of (±)-2-Methoxy-5,9-eicosadiynoic Acid (**2**)

#### 3.3.1. Nonadeca-4,8-diynal (**9**)

Aldehyde **9** was obtained as a white solid (mp 57–60 °C) in an 85% yield by refluxing dioxolane **4** (0.51 g, 1.60 mmol) in acetone/water (4.0 mL each) with HCl (conc) (1.60 mL) following the procedure outlined in Section 3.2.4.

IR (NaCl) ν_max_: 3311, 2954, 2924, 2852, 1691 (C=O), 1436, 1258, 1214, 917, 803, 723, 634 cm^−1^; ^1^H (CDCl_3_, 300 MHz) δ (ppm) 9.78 (1H, t, CHO), 2.62 (2H, dt, H-3), 2.48 (2H, t, H-11), 2.31 (4H, m), 2.13 (2H, t, H-4), 1.46 (2H, m), 1.27 (14H, brs, -CH_2_-), 0.89 (3H, t, -CH_3_); ^13^C (CDCl_3_, 75 MHz) δ (ppm) 201.0 (d, C-2), 81.3 (s), 80.1 (s), 78.7 (s), 78.5 (s), 42.8 (t), 31.9 (t), 29.58 (t), 29.55 (t), 29.3 (t), 29.2 (t), 29.0 (t), 28.8 (t), 22.7 (t), 19.4 (t), 19.3 (t), 18.7 (t), 14.1 (q, -CH_3_), 12.1 (t, C-4); GC/MS (70 eV) *m/z* (relative intensity): 274 (M^+^, 1), 273 (3), 259 (1), 217 (2), 175 (25), 161 (32), 148 (25), 147 (63), 133 (61), 131 (25), 119 (64), 106 (29), 105 (100), 95 (41), 91 (94), 81 (34), 79 (51), 67 (58), 55 (41); HRMS (APCI) Calcd for C_19_H_31_O [M + H]^+^ 275.2369, found 275.2369.

#### 3.3.2. 2-(±)-Trimethylsilyloxy-5,9-eicosadiynonitrile (**10**)

Nitrile **10** was obtained as yellow oil and in a 94% yield from the reaction of **9** (0.31 g, 1.12 g) with TMSCN (0.22 mL, 1.65 mmol) and catalytic amounts of Et_3_N in dry CH_2_Cl_2_ (10.0 mL) following the procedure outlined in Section 3.2.5. The product was used as such for the next step without further purification.

IR (NaCl) ν_max_: 2926, 2855, 2256 (CN), 1713, 1444, 1378, 1340, 1258, 1102, 1044, 944, 878, 722, 633 cm^−1^; ^1^H (CDCl_3_, 300 MHz) δ (ppm) 4.62 (1H, m, H-2), 2.32 (4H, m), 2.18 (2H, t), 1.95 (2H, m), 1.45 (2H, m), 1.25 (10H, brs, -CH_2_-), 0.89 (3H, t), 0.21 (9H, s, -OSi(CH_3_)_3_); ^13^C (CDCl_3_, 75 MHz) δ (ppm) 119.9 (s, C-1), 81.4 (s), 80.7 (s), 78.4 (s), 78.2 (s), 65.0 (d, C-2), 35.2 (t, C-3), 31.9 (t), 29.6 (t), 29.5 (t), 29.3 (t), 29.1 (t), 29.0 (t), 28.8 (t), 28.7 (t), 28.2 (t), 22.7 (t), 19.34 (t), 19.29 (t), 18.7 (t), 14.3 (q, -CH_3_), 14.1 (t, C-4), -0.49 (q, OSi(CH_3_)_3_); GC/MS (70 eV) *m/z* (relative intensity): 373 (M^+^, 6), 372 (8), 358 (29), 329 (10), 316 (9), 288 (24), 284 (15), 282 (32), 274 (30), 260 (26), 247 (38), 246 (62), 232 (29), 205 (16), 198 (21), 184 (28), 171 (15), 169 (24), 157 (32), 131 (22), 128 (34), 117 (25), 91 (34), 84 (79), 75 (42), 73 (100), 57 (57), 55 (44).

#### 3.3.3. Methyl (±)-2-Hydroxy-5,9-eicosadiynoate (**11**)

The methyl hydroxy ester **11** was obtained as colorless oil (61% yield for the two steps) from the reaction of **10** (0.39 g, 1.05 mmol) with concentrated HCl (6.2 mL) in 2-MeTHF (15.4 mL) followed by methanol esterification as detailed in Section 3.2.6.

IR (NaCl) ν_max_: 3501, 2926, 2855, 1740 (C=O), 1440, 1339, 1258, 1219, 1104, 998, 722, 667 cm^−1^; ^1^H (CDCl_3_, 300 MHz) δ (ppm) 4.32 (1H, m, H-2), 3.78 (3H, s, -CO_2_CH_3_), 2.31 (4H, m), 2.12 (2H, t), 1.97 (2H, m), 1.78 (2H, m), 1.44 (2H, m), 1.24 (14H, m, -CH_2_-), 0.96 (3H, t, -CH_3_); ^13^C (CDCl_3_, 75 MHz) δ (ppm) 175.4 (s), 81.3 (s), 79.9 (s), 79.3 (s), 78.5 (s), 69.2 (d, C-2), 52.6 (q, -OCH_3_), 33.4 (t, C-3), 31.9 (t), 29.6 (t), 29.5 (t), 29.3 (t), 29.1 (t), 28.96 (t), 28.8 (t), 22.7 (t), 19.5 (t), 19.4 (t), 18.7 (t), 14.6 (q, -CH_3_), 14.1 (t, C-4); GC/MS (70 eV) *m/z* (relative intensity): 334 (M^+^, 1), 291 (2), 275 (25), 257 (1), 245 (3), 235 (2), 221 (7), 208 (17), 195 (4), 175 (6), 161 (12), 155 (51), 147 (19), 133 (33), 121 (25), 119 (53), 105 (24), 95 (65), 93 (40), 91 (100), 90 (53), 81 (40), 79 (76), 77 (46), 67 (84), 55 (95); HRMS (APCI) Calcd for C_21_H_35_O_3_ [M + H]^+^ 335.2581, found 335.2579.

#### 3.3.4. (±)-2-Methoxy-5,9-eicosadiynoic acid (**2**)

The diynoic acid **2** was obtained as an oil in a 55% yield (for the two steps) in the reaction of **11** (0.05 g, 0.16 mmol), with NaH (0.01 g, 0.54 mmol) and methyl iodide (0.03 mL, 13.01 mmol) in dry THF (4.0 mL) followed by saponification with KOH/ethanol (1M) as described in Section 3.2.7.

IR (NaCl) ν_max_: 3500–2500 (-OH), 3310, 2926, 2855, 1723, 1443, 1258, 1207, 1119, 722, 637 cm^−1^; ^1^H (CDCl_3_, 500 MHz) δ (ppm) 3.98 (1H, m, H-2), 3.47 (3H, s, -OCH_3_), 2.33 (4H, m), 2.12 (2H, t), 1.97 (2H, m), 1.89 (2H, m), 1.46 (2H, m), 1.26 (14H, brs, -CH_2_-), 0.86 (3H, t, -CH_3_); ^13^C (CDCl_3_, 125 MHz) δ (ppm) 176.8 (s, C-1), 81.3 (s), 80.2 (s), 79.0 (d, C-2), 78. 6 (s), 78.5 (s), 58.7 (q, -OCH_3_), 31.9 (t), 29.6 (t), 29.5 (t), 29.3 (t), 29.2 (t), 29.0 (t), 28.8 (t), 22.7 (t), 19.43 (t), 19.38 (t), 18.7 (t), 14.7 (q, -CH_3_), 14.1 (t); HRMS (APCI) Calcd for C_21_H_33_O_3_ [M + H]^+^ 333.2435 found 333.2431.

### 3.4. *Ld*TopIB and *h*TopIB Inhibitory Assays

The *Ld*TopIB and *h*TopIB inhibitory bioassays were performed as previously described [7]. A brief description of the experimental procedure can be found as the header of Figure 1 and Figure 3.

### 3.5. Comparative Inhibition of Recombinant *Ld*TopIB by CPT, and Acids **1** and **2**

The experimental procedure employed can be found as the header of Figure 4 and the details of the preparation of *Ld*TopIB CPT^R^ (*Ld*TopIL^1−561/S175end^) were previously described in the literature [12,13].

### 3.6. Cytotoxicity of Acids **1**, **2**, and **12** towards *L. infantum* and *L. donovani*

The experimental procedures employed to assess the toxicity of acids **1** and **2** towards *L. infantum* and *L. donovani* are the same as the ones previously described [5,15]. CPT was used as the control drug.

## 4. Conclusions

The first total synthesis of **1** was achieved in nine steps and in a 5.9% overall yield. It was possible to corroborate both the structure and the *cis* double bond stereochemistry for the two double bonds in **1** with that of the natural fatty acid from the sponge *E. goffrilleri*. In addition, full spectral data is reported, for the first time, for the natural acid **1**. Both the natural acid **1** and its diynoic analogue **2** were good inhibitors of *Ld*TopIB with a mechanism different from that of CPT. Acids **1** and **2** did not display significant toxicity towards *L. infantum* promastigotes, but the synthetic acid **12** displayed a better toxicity towards *L. donovani* promastigotes, less toxicity towards murine macrophages, and therefore, a better therapeutic index. Therefore, it can be concluded that although a Δ^5,9^ diynoic α-methoxylated fatty acid displays a better inhibition of *Ld*TopIB than a Δ^6^ monoynoic α-methoxylated fatty acid with the same carbon chain length, the latter is more effective and selective towards *L. donovani* promastigotes.

## Supplementary Files

Supplementary File 1 3D structure (MOL, 2 KB)

Supplementary File 2 MOL-Document (MOL, 2 KB)
